# Perioperative Management of a Patient Taking Suboxone® at the Time of Ambulatory Surgery

**DOI:** 10.1155/2020/5628348

**Published:** 2020-03-09

**Authors:** Shawn H. Malan, Christopher H. Bailey, Narjeet Khurmi

**Affiliations:** Mayo Clinic Hospital, 5777 East Mayo Boulevard, Phoenix, AZ 85054, USA

## Abstract

In 2016, more than 11 million people reported misuse of opioids in the previous year. In an effort to combat opioid use disorder (OUD), the use of agonist/antagonist is becoming increasingly common, with more than 2.2 million patients reporting use of a buprenorphine containing medication such as Suboxone®. Buprenorphine is a unique opioid which acts as a partial *μ* agonist and *ĸ* antagonist. These properties make it an effective tool in treating OUD and abuse. However, despite its advantages in treating OUD and abuse, buprenorphine can make it difficult to control acute perioperative pain. We present a case in which the Mayo Clinic Arizona protocol for patients undergoing minimally invasive ambulatory surgery while taking Suboxone® is successfully executed, resulting in adequate postoperative pain control and timely discharge from the postanesthesia recovery unit.

## 1. Introduction

Buprenorphine is an opioid that acts as a partial agonist at the *μ*-opioid receptor and weak agonist at the *ĸ*-opioid receptor. It binds the receptor with high affinity with only partial agonism of the receptor, which prevents other opioids with full agonist properties from activating it. Each type of opioid receptor has different effects, so selective opioid receptor agonists have the advantage of avoiding some of the untoward effects caused by full opioid agonists (Table 1). When combined with naloxone, which is a mu antagonist that has poor oral absorption and undergoes extensive first pass hepatic metabolism, buprenorphine is a useful tool in discouraging opioid abuse [1]. If Suboxone® is melted and injected intravenously, the naloxone component will completely block the *μ*-opioid receptor for a time.

In 2016, the prevalence of prescription and heroin abuse was estimated at 17 million people worldwide, resulting in more than 33,000 deaths in the United States alone. This equates to, on average, 130 deaths every day in the United States [2]. In an effort to combat these addictions, the use of agonist/antagonist medications (e.g., buprenorphine/naloxone) is becoming increasingly common with more than 2.2 million patients reporting use of a buprenorphine containing medication such as Suboxone® [3].

**Figure 1:**
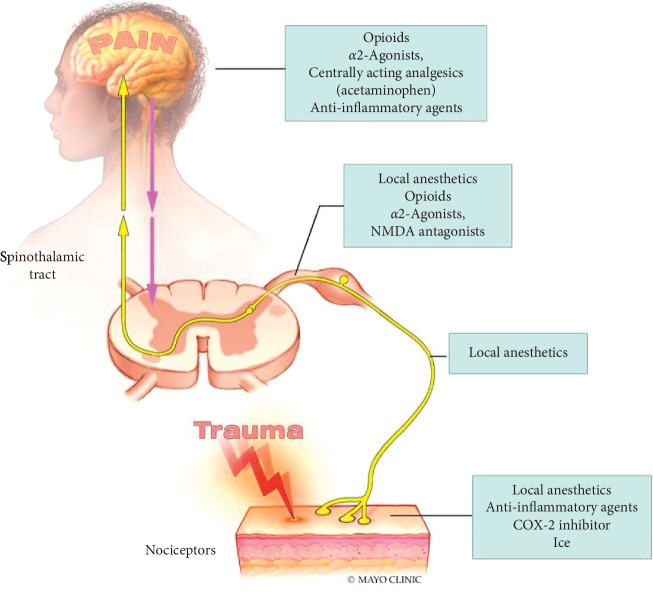
Analgesic sites of action.

**Figure 2:**
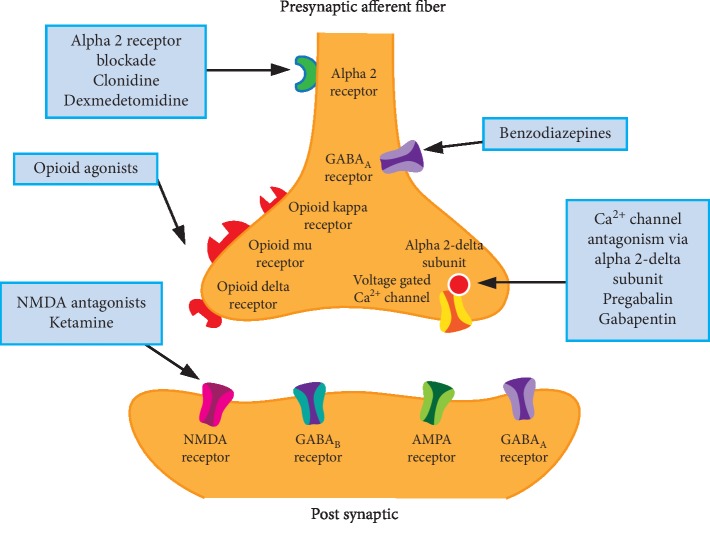
Receptors affecting pain transmission.

Buprenorphine is a useful tool in the fight against opioid addiction and abuse. However, due to its unique pharmacodynamics properties, it presents a unique challenge to anesthesiologists trying to treat acute pain in the perioperative setting [4, 5]. Although there is no consensus regarding optimal perioperative management of patients taking these medications, most strategies include a multimodal pain control approach with recommendations ranging from continuing buprenorphine and supplementing with full opioid agonists as necessary, to discontinuing buprenorphine preoperatively in exchange for full opioid agonists [6, 7]. Utilizing a multimodal approach to pain control allows for blockade of pain transmission at multiple sites along the nervous system as well as blockade of a variety of pain receptors with less dangerous side effects (Figure 1 and 2). Multiple case reports illustrate the unpredictability of pain control in these patients despite utilizing these strategies [8–10].

At the Mayo Clinic in Arizona, one of three strategies is typically employed (Table 2). The first strategy is to continue the patient's normal buprenorphine dose and employ a multimodal approach for acute perioperative pain control. This strategy is typically employed in elective, outpatient procedures. The second approach is to begin a slow wean of buprenorphine 1-2 weeks prior to the surgery with a goal to transition to a short acting, full opioid agonist (hydrocodone, oxycodone, etc.) 72 hours preoperatively. This strategy is recommended in the case of an elective procedure with planned postoperative admission. In the event that a patient presents for an urgent/emergent case while still taking buprenorphine, an immediate cessation of all forms of buprenorphine is recommended while starting a high dose, opioid based, patient controlled analgesia infusion which can be de-escalated as the buprenorphine wears off, which may take 72 hours or longer [11].

We present a case of an otherwise healthy patient presenting for a routine ambulatory surgery while taking an opioid agonist/antagonist preoperatively.

## 2. Case Presentation

A 37-year-old female (ASA II, 161 cm, 62 kg) with a 10-year history of chronic pelvic and abdominal pain on daily Suboxone® 2.9 mg (buprenorphine 2.9 mg/naloxone 0.71 mg) PO, prescribed to help treat her chronic pain, presents for a laparoscopic inguinal hernia repair under general anesthesia. Her medical history is significant for depression for which she takes Prozac. Her chronic abdominal pain began shortly after undergoing a total abdominal hysterectomy approximately 10 years previously. Since the time of the hysterectomy, she had undergone three open left inguinal hernia repairs. Additionally, she had undergone a right inguinal hernia repair, a salpingo-oophorectomy, a laparoscopic cholecystectomy, 6 laparoscopic surgeries for lysis of adhesions, and 2 cesarean sections. She was seen by multiple pain specialists who performed procedures including trigger point injections, nerve blocks, and radio frequency ablations—all of which offered some degree of transient pain relief. Pharmacologic therapies trialed to help manage her pain included ibuprofen, acetaminophen, duloxetine, gabapentin, muscle relaxants, oxycodone, and Suboxone® (buprenorphine/naloxone). While taking the Suboxone®, her pain varied from 4/10 to 6/10 on a numerical rating score. She has a remote, 2.5 pack-year smoking history. The patient's only allergy was to morphine which caused itching and fatigue. Preoperative physical exam was unremarkable aside from diffuse tenderness to palpation of her abdomen with her worst pain in the left lower quadrant. She rated her pain as 4/10 preoperatively.

Preoperatively, she received acetaminophen 1 gram PO, celecoxib 200 mg PO, gabapentin 300 mg PO, and dexamethasone 4 mg IV. She was given fentanyl 100 mcg IV and midazolam 2 mg IV in addition to lidocaine 100 mg IV, propofol 200 mg IV, and rocuronium 50 mg IV for induction. Her airway was secured with an endotracheal tube after an uneventful induction. Sevoflurane and dexmedetomidine infusion at a rate of 0.4-0.5 mcg/kg/hr were utilized for maintenance of general anesthesia. Intraoperative analgesics included ketamine 40 mg IV, fentanyl 200 mcg IV, and hydromorphone 0.6 mg IV during the 2-hour case. The surgeon infiltrated the surgical site with local anesthetic (0.25% bupivacaine mixed with liposomal bupivacaine) at the conclusion of the procedure. Her intraoperative course was unremarkable, and at the conclusion of the surgery she was extubated and taken to the postanesthesia care unit (PACU) with stable vital signs.

The patient arrived in the PACU at approximately 9:25 am and spent 3.5 hours there. During this time, she received fentanyl 100 mcg IV given in four 25 mcg boluses 15 minutes apart, Toradol 30 mg IV, and Tylenol 1 gram PO. All of these analgesics were given during the third hour of her PACU stay. She reported a pain score of 5/10 at the time of discharge which she was comfortable with.

## 3. Discussion

Evaluating whether a patient is a reasonable candidate to continue their agonist/antagonist medication perioperatively should be based on both patient specific and surgical factors. Continuing perioperative buprenorphine is a strategy that should be considered for surgeries that are not associated with significant postoperative pain. While it is reasonable to expect adequate analgesia with a multimodal approach, patients should be counseled beforehand not to anticipate a pain-free perioperative experience. Many patients taking agonist/antagonist opioids can benefit from continuing these medications perioperatively but, in particular, those being treated for OUD are at a high risk for relapse if buprenorphine is discontinued [12, 13].

Developing a strategy for perioperative pain control requires a pre-emptive, multimodal approach. Peripheral nerve blocks or neuraxial anesthetic techniques should be utilized whenever possible. Where these techniques are not possible, surgeons should infiltrate the incision with long acting local anesthetic. Higher than normal amounts of opioids will be required due to the antagonistic properties of these oral medications, and anesthesiologists should dose opioids appropriately in these patients. While pure opioid agonists are less effective in the setting of concurrent oral agonist/antagonist medications usage, they can still provide analgesia and should be used.

## 4. Conclusion

In our experience, it is reasonable and safe for select patients taking combined agonist/antagonist opioids to undergo ambulatory surgery without bridging to short acting pure opioid agonists. Evaluation to determine whether a patient is a candidate for this strategy should include multiple factors. These factors include type of surgery, dose of buprenorphine, reason for agonist/antagonist therapy (OUD treatment vs. chronic pain treatment), and access to postoperative care from pain specialists. Perhaps the most important of these factors is the original reason for treatment: chronic pain vs OUD. Strong consideration should be given to continuing perioperative buprenorphine in patients taking the medication for treatment of OUD due to the high rate of relapse that occurs when changing to a full opioid agonist in this population. Anesthesiologists, surgeons, and addiction medicine specialists should work closely to develop a multimodal approach specific to the patient in order to control acute postsurgical pain and decrease the likelihood of OUD relapse.

## Figures and Tables

**Table 1:** Opioid receptor characteristics.

Opioid Receptor	Site of Action	Effect
*μ* (*μ*1, *μ*2)	Brain Spine GI tract	*μ*1 (i) Supraspinal analgesia (ii) Physical dependence *μ*2 (i) Respiratory depression (ii) Physical dependence (iii) Miosis (iv) Euphoria (v) Reduced GI motility

κ	Brain Spine	Spinal analgesia Sedation Miosis Inhibition of ADH release

*δ*	Brain	Analgesia Euphoria Physical dependence

**Table 2:** Mayo Clinic Arizona periprocedural guidelines for patients taking buprenorphine.

Elective, outpatient procedure	Elective, inpatient procedure	Emergency procedure
*Preoperative guidelines*	*Preoperative guidelines*	*Preoperative guidelines*
(i) Consider anesthesia preoperative evaluation clinic consult (ii) Patient should continue taking buprenorphine as previously prescribed up until and on the day of surgery	(i) Consider anesthesia preoperative evaluation clinic consult 1-2 weeks prior to procedure: (ii) Complete cessation of buprenorphine by 72 hrs prior to procedure (iii) Slow taper protocol recommended: (1) Suboxone®, Subutex®, and Zubsolv®: decrease by 2 mg every 2-3 days, off at 72 hours prior to procedure (2) Butrans® patch: decrease by 50% 7 days prior to procedure, off at 72 hours prior to procedure (iv) Recommended preoperative analgesic dosing regimen: (1) Hydrocodone/acetaminophen 10–325 mg PO TID (dispense 9–15 tablets) (2) Begin 12 hours after last buprenorphine administration Day of procedure: (1) Recommendation to cancel procedure if noncompliant due to concerns with control of postprocedural pain. Reschedule and follow-up as appropriate	(i) Immediately stop all forms of buprenorphine (patch, oral) (ii) Anesthesia Pain Consult (iii) Start high dose IV PCA (iv) Decrease IV requirements as buprenorphine clears

*Intraoperative guidelines*	*Intraoperative guidelines*	*Intraoperative guidelines*
Maximize nonopioid adjuncts—NSAIDs, acetaminophen, local wound infiltration, and regional anesthesia Avoid use of additional opioid pain medications	Management similar to opioid tolerant patient Maximize nonopioid adjuncts—NSAIDs, acetaminophen, local wound infiltration, and regional	Management similar to opioid tolerant patient Maximize nonopioid adjuvants—NSAIDs, acetaminophen, local wound infiltration, and regional anesthesia Consider ketamine infusion Consider ICU admission for management of postoperative pain and monitoring

*Postoperative guidelines*	*Postoperative guidelines*	*Postoperative guidelines*
No additional opioid prescription given at discharge	Discharge with usual postoperative opioid course and resume buprenorphine once patient has been completely off opioids for minimum of 12 hours	Expect high dose PCA usage with goal for weaning during hospital course Discharge with usual postoperative opioid course and resume buprenorphine patient has been completely off opioids for minimum of 12 hours Recommendation for buprenorphine follow-up with prescribing physician.
